# The final plague outbreak in Scotland 1644–1649: Historical, archaeological, and genetic evidence

**DOI:** 10.1371/journal.pone.0306432

**Published:** 2024-11-13

**Authors:** Jenna Dittmar, Rebecca Crozier, Toni de-Dios, Christiana L. Scheib, Jackson W. Armstrong, Jenny Pape, Ross MacLennan, Ricky Craig, Marc Oxenham

**Affiliations:** 1 Department of Archaeology, University of Aberdeen, Aberdeen, Scotland; 2 Biomedical Affairs, Edward Via College of Osteopathic Medicine, Louisiana, Monroe, Louisiana, United States of America; 3 Institute of Genomics, University of Tartu, Tartu, Estonia; 4 Department of Zoology, University of Cambridge, Cambridge, United Kingdom; 5 Department of History, University of Aberdeen, Aberdeen, Scotland; 6 Aberdeen Archives, Gallery & Museums, Aberdeen, Scotland; 7 Independent GIS Specialist and Cartographer, Scotland; 8 School of Archaeology & Anthropology, Australian National University, Canberra, Australia; New York State Museum, UNITED STATES OF AMERICA

## Abstract

This paper has several aims: to determine if *Yersinia pestis* was the causative agent in the last Scottish plague outbreak in the mid-17^th^ century; map the geographic spread of the epidemic and isolate potential contributing factors to its spread and severity; and examine funerary behaviours in the context of a serious plague epidemic in early modern Scotland. Results confirm the presence of *Y*. *pestis* in individuals associated with a mid-17^th^ century plague pit in Aberdeen. This is the first time this pathogen has been identified in an archaeological sample from Scotland. The geographic spread of the plague from 1644 through to 1649 is generally, with some key exceptions, restricted to the central lowlands of Scotland. The role of administrative responses to the epidemic in managing its spread and distribution is unclear. Finally, normative funerary practices tended to co-exist with mass burial scenarios. In conclusion, the distribution of the epidemic is arguably a function of population density/distribution, transportation networks, and the chaos associated with the concurrent civil war. Administrative responses to the epidemic likely had a variable, albeit limited, effect in the central lowlands. More peripheral cities, such as Aberdeen, while also employing sophisticated plague prevention measures, were perhaps initially spared simply due to their distance from the central plague belt. It is unclear if a general fear of the dead and contracting the Pest from plague victims can be used to characterise mid-17th century Scottish public opinion. Mass burial appears to have been a practical approach to the logistical problems mass mortality presented, while many instances of normative burial treatment can also be seen.

## Introduction

The plague, often referred to as the Pest, entered Europe via Constantinople and port cities along the Mediterranean coast in 1347. Within a year, in early May 1348, it had reached the town of Weymouth in Dorset, southern England, by way of a ship travelling from Gascony, France [1]. It spread rapidly throughout Britain following heavily frequented coastal and riverine transport networks. Plague had a substantive foothold in north-eastern England by the first half of 1349 and had reached the north of England in the county of Durham in the summer of that year, while southern Scotland was suffering the effects of the Pest in the second half of 1349, ostensibly due to the return of Scottish troops that had been marshalling on the borders in Selkirk Forest before being infected [1, 2]. While sources are scant, by 1350 a third of the population of Scotland had apparently succumbed to the Pest according to the contemporaneous commentators John of Fordun (*Gesta Annalia II*) and Androw of Wyntoun [3–5]. The Orkney and Shetland Islands, at the time under Norwegian control, also appear to have also been visited by the Black Death in 1348 or 1349 [1] although little is known of its effects.

Scotland was subsequently affected by numerous visitations of the Pest over the following centuries through to the final major outbreak, in Scotland at least, in the mid seventeenth century. Relatively good historical records covering this period, in addition to the identification of the remains of potential plague victims of this specific visitation, provide an opportunity to explore the final throes of a centuries old reoccurring epidemic in Scotland. In this context, the aims of this paper are fivefold: (1) determine if the bacterium *Yersinia pestis* was responsible for the plague outbreak in Aberdeen in 1647–48, and by implication was likely associated with the general spread of plague in Scotland between the years 1644 and 1649; (2) map the spread of the mid-17^th^ century Scottish outbreak in order to explore potential vectors and ecologies of dissemination of this deadly infectious disease; (3) discuss conditions within Scotland that may have contributed to the spread and severity of the epidemic; (4) explore the role or implications of administrative interventions (e.g., restriction of freedom of movement) may have had on the spread and/or severity of outbreaks; and (5) in this context discuss treatment and disposal of those that had died from the Pest.

## Methods and materials

Our methodological approach combines historical, human osteoarchaeological, and ancient DNA (aDNA) techniques. Human remains were identified in the collections of Aberdeen Art Gallery and Museums that were excavated in an industrial compound at York Place, Aberdeen, in 1987 [6, 7]. The site in question is believed to be associated with the general location of plague pits that were established on the Queen’s Links (a Scots term for coastal sand dunes) during the 1647–8 outbreak of the plague. An earlier excavation in the same general area for the purpose of constructing a new sewer in 1891 recovered skeletal remains also believed to be associated with the 1647 plague pits [8]. This small assemblage consisted of a minimum number of four individuals (based on the presence of frontal bones). With respect to ethical oversight, written permission was obtained from the custodians and curators of the human archaeological material, the Aberdeen Art Gallery and Museums, Aberdeen Treasure Hub, Scotland, for destructive sampling for the purpose of C14 dating and aDNA analyses.

The remains from the 1987 excavation were C14 dated to determine if they did indeed belong to the mid seventeenth century. A tooth from each of three individuals, a fourth individual did not have preserved teeth, was sampled for ancient DNA analysis (see Table 1 for details) and sent to the University of Tartu, Institute of Genomics (UTIG). Inside the dedicated clean lab tooth roots were removed using a sterilised steel drill wheel, decontaminated with 6% w/v bleach (NaOCl), rinsed thrice with purified water (Millipore) and finally with 70% ethanol before being dried under UV light inside a class IIB safety cabinet. Samples were processed in the clean room of the dedicated ancient DNA laboratory of the Institute of Genomics, University of Tartu, Estonia following established published protocols detailed on protocols.io. Sampling: [9], decontamination: [10], extraction and purification: [11], Double-stranded, dual-indexed libraries were produced following: [12]. DNA was sequenced using the Illumina NextSeq500/550 High-Output 150 cycle kit in paired-end mode. [see SI Additional Sample Information in S1 File].

**Table 1:** York place sample.

Museum No.		Osetological	aDNA	DNA tooth	δ 13 C	δ 13 N	% marine	14C Age	C14 date (CE)	C14 date (CE)	C14 date (CE)
ABDMS	Age-at Death	sex	sex	sample			diet*		Calibrated (95.4%)	Calibrated (95.4%)**	Calibrated (68.3%)**
23509.1	Adult	F	F	13	-19.3	13.3	20	398±28	1440–1624	1485–1659	1520–1644
23509.2	Adult	M	M	47							
23509.3	Preadult	?	M	46	-19.6	13.3	16.5	320±25	1491–1643	1522–1950	1530–1797
23509.4	Adult	F	N/A	N/A	-19.7	13.2	15.3	359±24	1458–1633	1499–1664	1524–1653

13 = right maxillary canine; 47 = right mandibular second molar; 46 = right mandibular first molar

* Estimated using Arneborg [70]

** using marine correction

Dates calibrated using calib 8.20

Marine20 ΔR = -133

Uncertainty in Marine20 ΔR = 36

The generated raw data was returned in the form of paired FASTQ files. Sequencing adapters were removed and collapsed pair reads were collapsed using AdapterRemoval2 [13]. Low complexity sequences that are insufficiently specific to randomly match a wide range of different microbial genomes (i.e. regions composed by few repeated nucleotides), were removed using prinseq [14]. Finally, all duplicated reads were collapsed using bbmap tool suite [15]. All reads were processed with KrakenUniq and the standard MicrobialDB [16]. To assess the validity of the microbial assignations generated by KrakenUniq, an E-value was applied. In brief, this value identifies if the sequence reads match a specific region of a microbial genome, behaviour corresponding to an erroneous match to a generally conserved region, or in contrast, are randomly distributed across a genome, which should be expected with a genuine match [17]. Potential positives were further validated by mapping them against *Y*. *pseudotuberculosis* and *Y*. *pestis* reference genomes using BWA backtrack [18], with a relaxed edit distance (-n 0.01) and seeding disabled (-l 65536), to account for aDNA damage [19]. We quantified the number of mapped sequence reads with a mapping quality of 30, and their edit distance, to evaluate if they were closer to *Y*. *pseudotuberculosis* or *Y*. *pestis*. We also characterised the presence of aDNA associated damage in the reads using MapDamage2 [20]. Further validation of the sequence reads was carried out using BLAST [21].

Historical evidence for the presence of plague, and its effects, were collected from a range of historical sources. Specifically, we were interested in recording geographic locations, often identified to the parish level, dates (usually stated as a season, but sometimes as a month or range of months) of infection, severity of the outbreak if recorded and any information relating to administrative interventions, such as restricting freedom of movement or hygiene measures that may have been implemented.

## Results & discussion

### 1. Identification of *Yersinia pestis* in Scotland

As noted, an assessment of the disarticulated assemblage of human skeletal remains recovered from York Place contained a minimum number of four individuals (based on presence of frontal bones); three adults and one preadult (<18 years of age). The osteological characteristics of the cranial remains indicate two adult females and one possible male. Biological sex was confirmed genetically for two of these individuals and the preadult was determined to be male.

**Fig 1:**
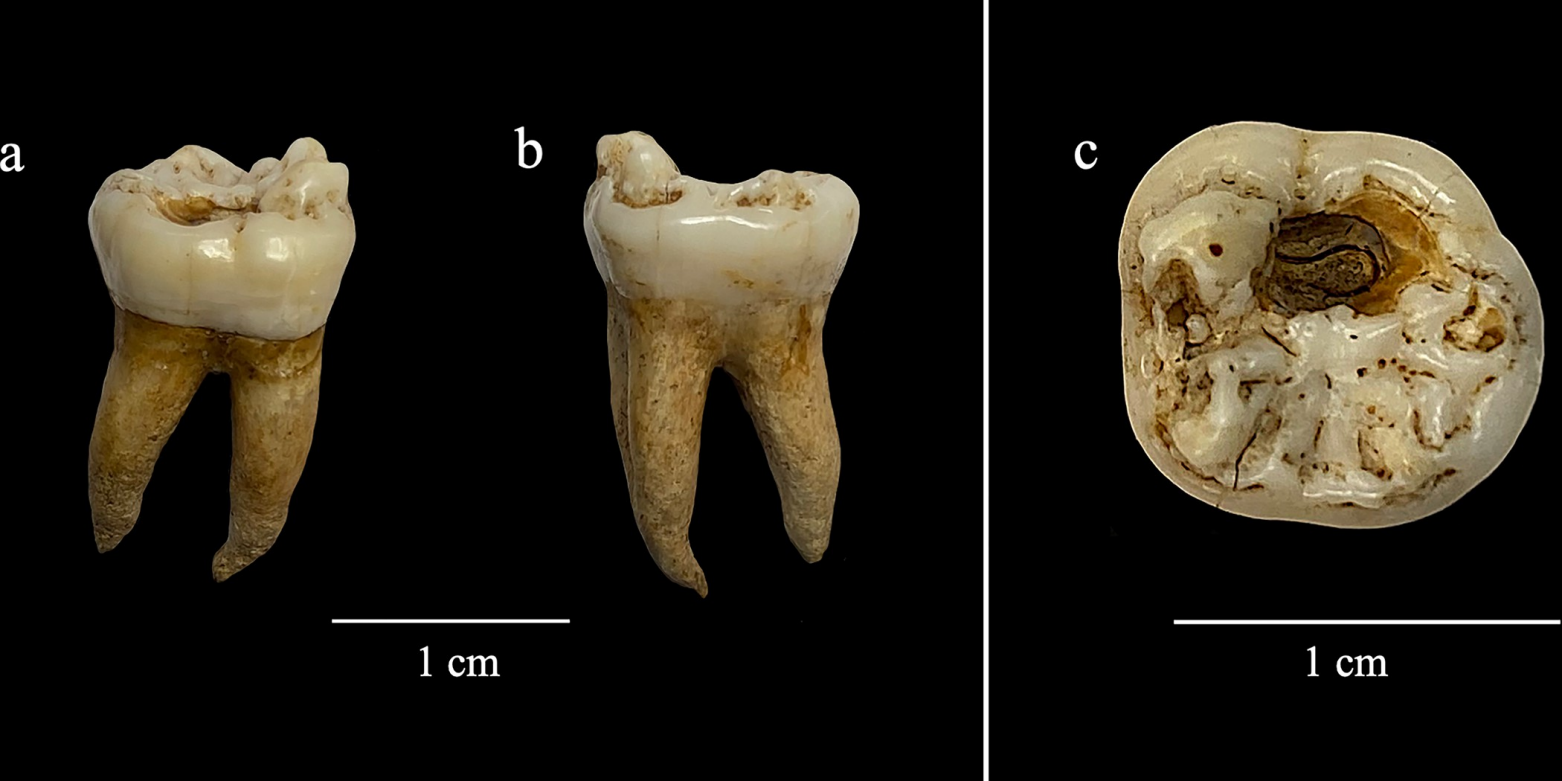
Mandibular right first molar: (a) buccal view, (b) lingual view, (c) occlusal view. The complex morphology of pseudo-cusps and ridges are due to interruptions to enamel formation during growth (enamel hypoplasia) and are commonly referred to as Fournier or mulberry molars (see [23]).

Of note is the preadult individual which is estimated to be 12.5 years old based on dental development [22]. The anterior portion of the mandible is present, from the second premolars forward. A ‘mulberry’ molar (Fig 1), the right first, is the only preserved molar in the mandible. This tooth displays a small occlusal carious lesion. The presence of a mulberry molar is suggestive of congenital syphilis [23, 24], although aDNA analysis failed to isolate *Treponema pallidum* in this sample. One instance of the co-occurrence of plague and treponemal disease has been recorded for fifteenth century Lithuania [25], another in early 18^th^ century Estonia [26], with this potentially being the first case known for Britain.

Results of C14 dating, including various calibrations, are provided in Table 1. The dates are consistent with an expectation that these individuals died in 1647/8. Indeed, they cannot be later than 1650 as they each tested positive to *Yersinia pestis*, and plague did not revisit Scotland in any substantive manner after 1649 [27 (p. 186)].

The presence of *Y*. *pestis* was identified in each of the three screened samples (YYY054, YYY055, YYY056 [see SI Metagenomic Screening Results in S1 File]. After mapping against *Y*. *pestis* and *Y*. *pseudotuberculosis* reference genomes (Table 2) [see SI Reference Genomes Used in S1 File], all the hits for *Y*. *pestis* display a lower edit distance to this particular bacterium than to *Y*. *pseudotuberculosis* [see SI Edit Distance Comparison in S1 File], mitigating against an infection with the later (a possibility due to their extremely close genetic proximity). YYY054 and YYY055 edit distance distributions show a slight shift to higher values, a fact that could be explained by some cross assignation with other bacteria present in the metagenomic mixture. Regarding damage, only YYY056 has enough sequence reads to find an aDNA damage related signal [see SI aDNA Damage Patterns in S1 File], since this procedure requires a critical number of retrieved sequence reads to be accurate. Despite the low resolution, and given the patterns found in the three samples, the damage is similar to that expected in genuinely ancient samples with a low number of reads.

**Table 2:** *Yersinia pestis* and *Yersinia pseudotuberculosis* mapping statistics^1^.

Sample	Species	Mapped Q30 reads	Av. Length Bp	Av. Edit Distance	Av. Damage 5’	Av. Damage 3’	Pseudotuberculosis Complex Blast	Exclusive Blast Reads
YYY054	*pestis*	100	37.34	1.16	4.17%	7.13%	41	6
YYY055	*pestis*	92	37.33	1.39	7.63%	1.39%	30	3
YYY056	*pestis*	260	44.3	0.8	13.09%	6.1%	179	11
YYY054	*pseudotuberculosis*	101	37.68	1.24	4.14%	7.37%	41	1
YYY055	*pseudotuberculosis*	89	37.29	1.5	6.66%	4.96%	30	0
YYY056	*pseudotuberculosis*	253	44.86	0.92	11.82%	7.35%	179	0

1 Mapping statistics of sequences mapped against *Yersinia pestis* and *Yersinia pseudotuberculosis* reference genomes. Those include mapped reads with quality 30, average read length, average edit distance, average deamination on the 3 last bases at 5’ end, average deamination on the 3 last bases at 3’ end, positive reads against *Y*. *pseudotuberculosis complex* using BLAST, and species’ exclusive reads assigned by BLAST.

Finally, additional BLAST screening of the mapped reads further confirms the authenticity of the samples, with most of the reads assigned unequivocally to *Y*. *pestis* and to the *Y*. *pseudotuberculosis* group to which *Y*. *pestis* is a part (YYY054 = 41, YYY055 = 30, YYY056 = 179). Interestingly, YYY054 and YYY055 also show a substantial fraction of sequence originating from widely conserved bacterial sequences or environmental organisms. In conclusion, despite the presence of a limited number of environmental sequence reads, we can confirm the presence of *Y*. *pestis* in all three samples (YYY054, YYY055 and YYY056) (Table 2).

### 2. The spread of the 17^th^ century Scottish epidemic

**Fig 2:**
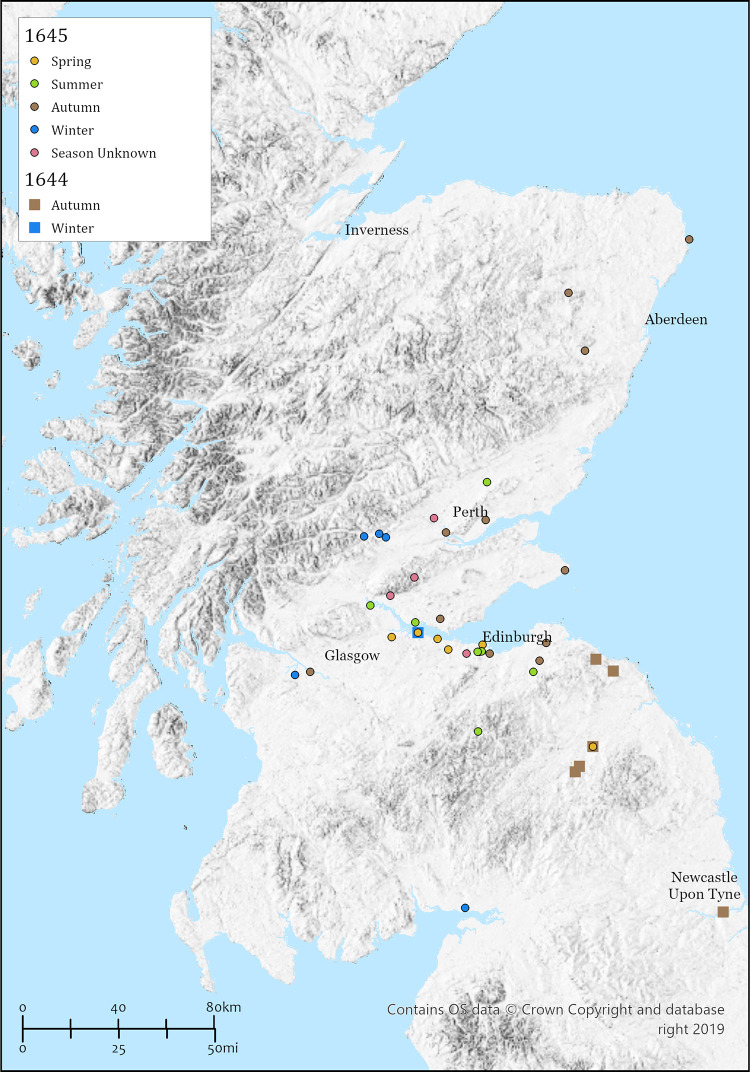
Distribution of plague outbreaks between 1644 and 1645 (see also Table 3). [OS. "Topographic" [basemap]. Scale Not Given. "GB Topographic". October 9, 2023. https:\\tiles.arcgis.com/tiles/qHLhLQrcvEnxjtPr/arcgis/rest/services/GB_Basemap_v1/VectorTileServer].

Figs 2 (1644–45), 3 (1646–47) and 4 (1648–49) (see also Table 3) provide a graphic representation of the epidemic of the 1640s in Scotland. The geographic origin(s) and initial means of entry of the final plague epidemic to affect Scotland in the mid-seventeenth century is unclear, although (reminiscent of the ostensible association with returning soldiers and the first Scottish outbreak in 1349) an initial foothold by infected Scottish soldiers returning from the siege of Newcastle (which ended on the 19^th^ of October 1644) has been suggested [28, 29]. The following account of the spread, including timing and severity, is based upon work by Flinn and colleagues [27] unless otherwise referenced.

**Fig 3:**
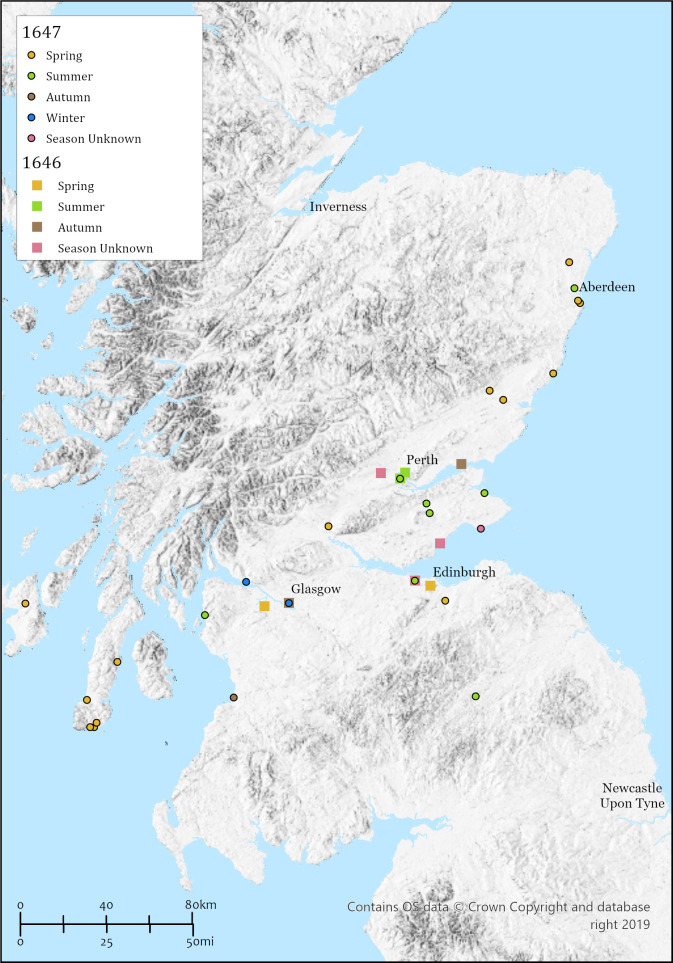
Distribution of plague outbreaks between 1646 and 1647 (see also Table 3). [OS. "Topographic" [basemap]. Scale Not Given. "GB Topographic". October 9, 2023. https:\\tiles.arcgis.com/tiles/qHLhLQrcvEnxjtPr/arcgis/rest/services/GB_Basemap_v1/VectorTileServer].

**Fig 4:**
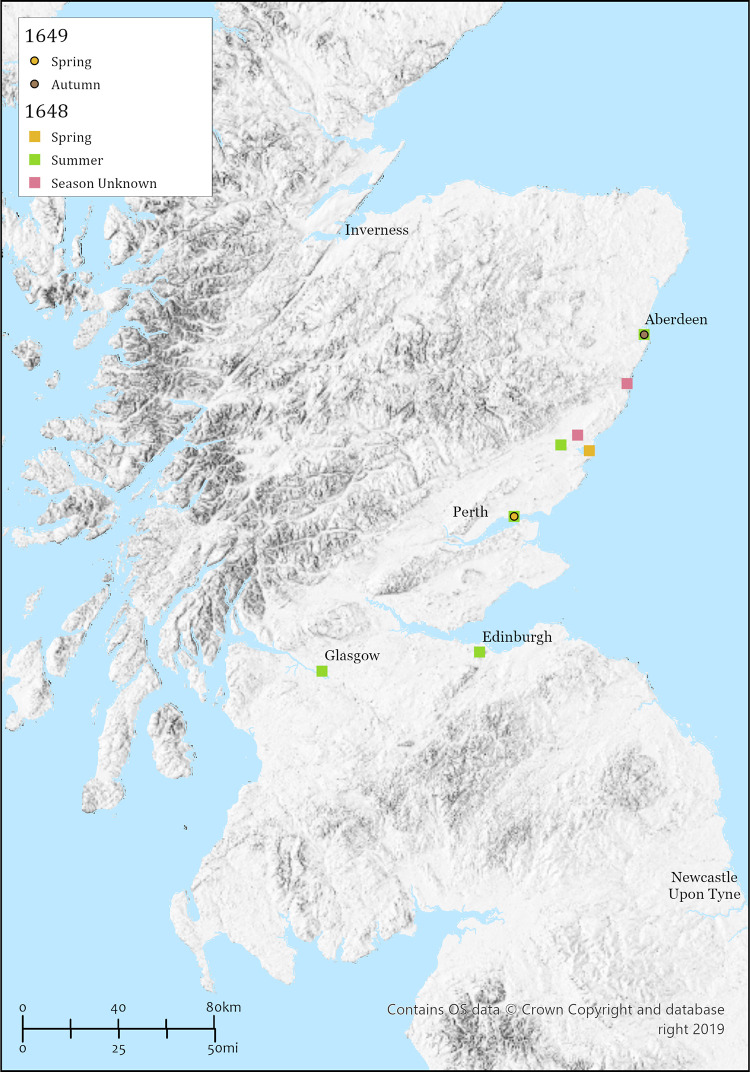
Distribution of plague outbreaks between 1648 and 1649 (see also Table 3). [OS. "Topographic" [basemap]. Scale Not Given. "GB Topographic". October 9, 2023. https:\\tiles.arcgis.com/tiles/qHLhLQrcvEnxjtPr/arcgis/rest/services/GB_Basemap_v1/VectorTileServer].

**Table 3:** Distribution of plague outbreaks between 1644 and 1649 (see Figs 2–4).

town/city/parish	1644	1645	1646	1647	1648	Reference	town/city/parish	1645	1646	1647	1648	1649	Reference
New Castle	Autum					[27 (p. 136–7)]	Fife						
**Scottish borders**							Culross	Summer					[27 (p. 141)]
Oldhamstocks	Autum					[27 (p. 137)]	Dunfermline	Autum					[27 (p. 141)]
Grantshouse	Autum					[27 (p. 137)]	Dysart		season uk				[27 (p.141), 45 (p.165-6)]
Bonjeward	Autum					[27 (p. 137)]	Crail	Autum					[27 (p. 141)]
Nisbet	Autum					[27 (p. 137)]	St Andrews			Summer			[27 (p. 145)]
Kelso	Autum	Spring				[27 (p. 136)]	Falkland			Summer			[27 (p. 145)]
Peebles		Summer				[27 (p. 140), 42 (p. 379)]	Elie			season uk			[27 (p. 145)]
Annan*		Winter				[27 (p. 142)]	Auchtermunchty			Summer			[27 (p. 145)]
Ashkirk				Summer		[27 (p. 145)]	**Perthshire**						
**Ayrshire**							Perth	Autum	Summer	Summer			[27 (p. 141–2, 144)]
Ayr				Autum		[27 (p. 144), 35 (p. 277)]	Scone		summer				[27 (p. 142)]
Largs				Summer		[43 (p. 786)]	Moneydie	season uk					[46 (p. 200)]
**Edinburgh &**							Methven		season uk				[27 (p. 142)]
**Midlothian**							Crieff	Winter					[27 (p. 141)]
Edinburgh		spring	spring		Summer	[27 (p. 135, 139–40, 146)]	Monzievaird	Winter					[27 (p. 141)]
Crammond			season uk	Summer		[27 (p. 143, 145)]	Glendeven	season uk					[27 (p. 141)]
Leith		spring				[27 (p. 137–8)]	Comrie	Winter					[27 (p. 141)]
Cannongate		Summer				[27 (p. 138–9)]	Meigle	Summer					[27 (p. 141)]
St Cuthberts		Summer			Summer	[27 (p. 139, 146)]	Dunblane			spring			[27 (p. 144)]
Duddingston		Autum				[27 (p. 135, 140)]	**Argyll**						
Corstorphine		season uk				[43 (p. 220)]	Carradale			Spring			[27 (p. 144)]
Dalkeith				Spring		[27 (p. 145), 43 (p. 486)]	kilkivan			Spring			[27 (p. 144)]
**Eastlothian**							Kilblaan			Spring			[27 (p. 144)]
Saltoun (Gilchriston)		Summer				[27 (p. 140), 44 (p. 310)]	Dunaverty			Spring			[27 (p. 144)]
Bolton		Autum				[27 (p. 140)]	Kilcolmkill			Spring			[27 (p. 144)]
Athelstaneford		Autum				[27 (p. 140)]	Islay (Island)			Spring			[27 (p. 143)]
**Westlothian**							**Angus &**						
Kirkliston		spring?				[27 (p. 141)]	**Kinkardineshire**						
Abercorn		spring				[27 (p. 140)]	Dundee		Autum		Summer	spring	[27 (p. 143, 146)]
Bo’ness	Winter	spring				[27 (p. 137, 140), 28 (p. 58)]	Inchture	Autum					[27 (p. 141)]
**Glasgow &**							Brechin			Spring	summer		[27 (p. 144)]
**Lanarckshire**							Montrose				spring		[27 (p. 146)]
Glasgow			Autum	winter	summer	[27 (p. 143–4, 146)]	Menmuir			Spring			[27 (p. 144), 47 (p.656-7)]
Govan		Autum				[27 (p. 142)]	Logie Pert				season uk		[27 (p. 146)]
Paisley		winter	Spring			[27 (p. 142), 32 (p. 254)]	Inverbervie			Spring			[27 (p. 145)]
**Stirlingshire,**							Stonehaven				season uk		[27 (p. 146)]
**Clackmannanshire,**							**Aberdeenshire**						
**& Dumbartonshire**							Old Machar			Summer			[27 (p. 135)]
Stirling		Summer				[27 (p. 141)]	Raemoir	Autum					[27 (p. 141)]
Falkirk		Spring				[27 (p. 141)]	Licklyhead	Autum					[27 (p. 141)]
Alva		season uk				[31 (p. 201)]	Peterhead	Autum					[27 (p. 141)]
Dumbarton				winter		[27 (p. 143)]	Pettymuck			Spring			[27 (p. 145)]
							Torry			Spring			[27 (p. 145)]
							Aberdeen			Spring	summer	Autum	[27 (p. 145–6)]

* Annan selected to represent the diverse parishes affected this year

Season: Spring-March to May; Summer-June to August; Autum-Sept to Nov; Winter Dec to Feb

Whatever the role of returning soldiers, cases of plague were recorded from the Scottish border in both Berwickshire and Roxburghshire, the latter on the 27^th^ of October or eight days after the end of the Newcastle siege (see Fig 2). Indeed, the 1644 outbreak occurred during the autumn and winter months. It is worth noting that plague was reported in the parishes of Bonjeward and Nisbet, which are approximately 60 miles northwest of Newcastle. It is possible plague was also present in Kelso, some 40 miles southeast of Edinburgh, at a similar time. The only other reference to plague in 1644 is for Bo’ness on the 17^th^ of December, which is some 60 miles northwest of Kelso or 20 miles west of Edinburgh. Given the lack of any other evidence for plague in any other parishes in 1644, including Edinburgh, the Bo’ness outbreak may have been ship borne.

The following year, 1645 (Fig 2), saw widespread and serious outbreaks of plague which would appear to start in the spring. The furthest southern parish with plague is Kelso in Roxburghshire, while a little further north there are serious outbreaks in Edinburgh by late March/early April, not abating until the winter. The Port of Leith, just two miles from central Edinburgh, had cases in April/May, again not subsiding until toward the close of the year. Stoakley and colleagues [30] provide a detailed account of the devastating effects of the pestilence in Leith, which claimed the lives of over 2,500 individuals. Bo’ness seems to see another visitation during the spring/summer [28], while a single plague death in Abercorn, only seven miles east of Bo’ness, likely also occurred in the spring although no month is given. Eight miles west of Bo’ness, a serious outbreak at Falkirk, lasting from April through to September has been recorded. Flinn and colleagues [27 (p.141)] refer to a “good many” dying in Alloa when mentioning a single suspected case in Tillcoultry, however, the reference to Tillicoultry [31 (p. 201)] notes “a good many persons died of it [plague] in Alva”, a small parish less than three miles to the west of Tillicoultry. Further west, it is present in Govan, five miles west of downtown Glasgow, in early March and appears to have become more serious during the autumn. Paisley was particularly badly affected towards the close of 1645 continuing to the middle of the following year [32].

The summer saw an expansion of infection into parishes close to central Edinburgh including St Cuthberts and Cannongate, the latter experiencing very high mortality. It is possible Corstorphine was also affected, severely, at this time [see 33 (p. 56)]. Further afield, serious summer outbreaks in Stirling and Culross, Fife, are documented. Gentles [34] notes that Parliament had relocated to Perth in the summer due the plague having spread from Edinburgh and then onto Stirling. By autumn other parishes in Fife were affected, with an occurrence in Crail. Duddingston, just east of Edinburgh, saw an autumn peak of the infection while East Lothian also saw autumn outbreaks. To the north, Perth was visited in autumn as was Inchture in Angus while by November Aberdeenshire had several outbreaks. In December 1645 plague was noted in parishes west of Perth and as far south as Annandale in Dumfriesshire.

The following year, 1646 (Fig 3), saw a restricted distribution of plague, with some cases reported close to Edinburgh in the spring, and some documented instances in August. The exception to the rule was a very severe outbreak during the summer in Perth with a mortality of some 3,000 individuals. It is also worth noting some apparent evidence for plague in Glasgow toward the end of the year. Cases would seem to continue into the new year and throughout much of 1647, until August at least. Flinn and colleagues [27] are inclined to the view that we may be seeing a typhus outbreak and or co-infection with plague among Major-General Middleton’s soldiers whose arrival coincided with the initial but later 1646 cases. They [27] make a similar case for the several soldiers that apparently died in January/February 1647 of the Pest in Dumbarton, some 20 miles northwest of Glasgow. Either way, infection in Glasgow increased in January/February of 1647 and continued, albeit apparently with low mortality, through to August.

Plague continued in Midlothian with reports for Dalkeith, seven miles southeast of Edinburgh, in mid-April 1647. To the west and south a serious outbreak was seen during the summer in Largs, Ayrshire, while at least 34 individuals succumbed in Ayr itself between September and November [35]. The Pest continued for its third year running in Perth where it had apparently spread to the garrison. St Andrews seems to have had some presence of plague in the summer, while the odd case was reported in other parishes in Fife. Further north in Angus, April outbreaks were seen in Inverbervie, Menmuir, and Brechin, the latter parish being particularly badly affected with some suggestion of as many as 600, or two thirds of the population, dying of plague within a four-month period [36 (p. 89), 27 (p. 144)]. However, the original petition on the 23^rd^ of May 1649 refers to “…the tuo pairt of the inhabitants…” [37 (1649/5/121)], which, following Dictionaries of the Scots Language the term “part” (entry for 6 (b)) indicates the most probable translation of ‘the tuo pairt’ as ‘the half part’, or in other words half of the inhabitants, rather than two thirds, died of the plague, which is still a considerable number of victims.

The apparent outbreak of plague, with ostensibly high associated mortality, in the Kintyre peninsular in 1647 seems somewhat anomalous at face value but is perhaps associated with troop movements. Troops under the command of Major-General Middleton and Lieutenant-General Leslie had travelled and fought together against the Royalists in 1646 with Middleton later being garrisoned in Glasgow (see above), while Leslie headquartered at Dunblane prior to marching to the Kintyre peninsular [29]. While Dunblane may have been free of plague it was present in the immediate area, with Leslie leaving Dunblane on the 17^th^ May and making it to Inverary on the 21^st^ of May [29]. Engagements with Royalists were made at Kilcalmonell, the battle of Rhunahaorine Point, after which Leslie travelled further south to Lochhead (Campbeltown) arriving on the 25^th^ of May, and then onto Dunaverty castle by the end of May. It is here that, following a short siege, some 300 individuals are reported to have been executed, after which Leslie heads to Islay, landing on the 25^th^ of June, in pursuit of the remaining Royalists and took Dunnyveg castle on the 5^th^ of July [29].

These troop movements show the speed and extent of Leslie’s travel through the peninsular and onto Islay, although a causal link between this campaign and plague, even its very existence, is lacking. Mckerral [29] relays anecdotal evidence for the spread of the Pest throughout the Kintyre peninsula in the mid to late 1640s, although there is firmer evidence in the form of parish records for Kilcomkill and Kilblaan, at least, indicating significant abandonment of landholdings around this time, suggesting substantive local mortality.

An entry in the Council Register of the Burgh of Aberdeen on the 12^th^ of April 1647 states “…the magistratis of this burghe hade received certaine intelligence that the plague of pestilence was raging in [Inver]Bervie…” [38 (p. 81)]. Aberdeen, 26 miles north of Inverbervie, was steeling itself for the return of the plague which had spared the city since the last outbreak almost 100 years previously in 1545 [39]. Reports of the Pest in Pettymuck, 12 miles northwest of Aberdeen, and even Tory, just across the River Dee from New Aberdeen, were followed by a cessation of Aberdeen Council meetings on the 26^th^ of May. The 1647 outbreak appears to have lasted until December of that year, returned in the summer of 1648 with a final appearance in late 1649 [27]. The death toll was some 1600 individuals with an additional 140 deaths within Futtie and Tory [40 (Vol. 1, p. 271)], while 20 died of the Pest in Old Machar [27 (p. 145)] or Old Aberdeen.

While not as geographically widespread, the Pest re-emerged, and with a vengeance in some areas, in 1648 (Fig 4). It either continued or re-emerged in the summer in Edinburgh, including St Cuthberts, while Glasgow was afflicted for the third year in a row with infections increasing by July and peaking in August, apparently the worst year of plague the city had seen [27]. In Angus there were severe outbreaks in Montrose apparently serious enough to close the Kirk sessions from May through to February in the following year 1649. It re-emerged in Brechin during the summer while a serious outbreak was seen in Dundee in August that lasted six months. Further north, it apparently raged in Logie Pert, some seven miles northwest of Montrose, while a single case was recorded at Stonehaven in Kinkardineshire. As for Aberdeen, the Pest either returned or re-emerged during the summer of 1648. Except for a few cases in Dundee in March of 1649 (possibly a persistence of the previous year’s outbreak) and cases in October of the same year in Aberdeen, the Pest had finished with Scotland until a minor outbreak in Glasgow in 1900 [41].

### 3. Conditions for the transmission of the pest

There are three forms of plague, the two most common being bubonic, where the bacterium *Y*. *pestis* is transmitted to a human host by way of an infected rat with a flea being an intermediary vector; and the pneumonic form where aerosolized bacteria infect the lungs of the human host. A third form is septicaemic where the blood stream is infected, usually secondarily to the bubonic or pneumonic forms. It has, however, been plausibly argued that the pathogen can be transmitted by way of human ectoparasites, thus obviating the need to rats or rat borne fleas [48]. Moreover, it has been noted that pneumonic transmission can occur in 20% of bubonic outbreaks [48 (p. 1308)], which will likely add to the complexity of mortality patterns in the past.

As mentioned, there has been some suggestion that the plague was transmitted by troops, with the initial visitation in late 1644 attributed to the victorious return to Scotland of the Covenanter army following the siege of Newcastle and via the movements of Leslie’s Covenanter troops through Kintyre and Islay in 1647. While the physical movement of infected troops, or any infected individual for that matter, would increase the risk of transmission between towns and villages, direct evidence for the role of troop movements and the spread of plague is lacking. Notwithstanding, it is worth noting the general devastation caused by the civil war in Scotland particularly between 1644 and 1648.

Gentles [34 (p. 436–7)] estimates an overall mortality rate of 7% for the Civil war period (1640–52), with the Scottish rate being approximately 6%, or some 25,000 battle deaths and a further 35,000 deaths attributable to accident or disease (it is not clear how many, if any, of these disease related deaths were attributable to the plague or if this refers to other common war related diseases of the time such as typhoid and typhus). The battle deaths alone are similar to Flinn et al.’s [27] estimates of plague mortality (20–30,000 individuals) for Scotland during the same period (see [34 (p. 435)]). Added to war figures is an estimate of over 6,000 Scots dying on English soil, 10,000 Scots soldiers taken prisoner never to return home [49 (p. 212)], as well as a conservative estimate of an additional 30,000 wounded soldiers [34 (p. 436)], many of whom would have taken debilitating injuries to their eventual deaths. The economic costs in terms of lost labour and increased care (and financial) burdens on the families of these surviving combatants would have been considerable. These warfare-related mortality and morbidity figures aside, there was additional enormous disruption in terms of famine and loss of shelter.

The effects of the conflict, laying waste to both townships and agricultural land in the central and southwestern Highlands, created a large starving refugee population that could only be addressed by way of considerable amounts of food aid being transported into the region in 1646 [34]. The east coast was not spared with the Royalist Army under Montrose sacking Aberdeen on the 13^th^ of September 1644. Carlton [49 (p. 177, 259)] argues this resulted in over a thousand deaths, rampant looting, and atrocities such as rape. Gentles [34 (p. 238–9)], on the other hand, suggests over 500 were slain and also questions reports of mass rapes in the city. Firsthand accounts state that “many of the Fyff regiment [Covenanter Fifth] wer killed…” with “many [townsmen] mae to the number near of aucht scoir [eight score, or 160] …for the enemie…did kill all, old and young, whom they fand on the streittes…” [38 (p. (29)]. By any account, even 160 civilian deaths is an appalling toll with the associated sacking of the town being felt for many years to come.

It is worth looking at the much smaller town of Brechin, some 40 miles south of Aberdeen and 8 miles directly west of Montrose. By virtue of its position on the main north-south road, and its significance as a cathedral site, Brechin was occupied by troops on numerous occasions particularly between late 1644 and late 1645 [36 (p. 82–86), 50 (p. 66–68)]. As if supplying food and shelter to occupying troops were not enough, in March 1645 Montrose’s troops, in response to the townspeople hiding their goods and fleeing the town, “found their goods, plundered the castle and haill town, and burnt about 60 houses” [51 (p. (286)]. Over a hundred years later the population of Brechin was reported as 3,181 [52 (p. (459)] and even assuming a slightly smaller population in 1645, this amounts to a substantive degree of destruction and displacement of the population.

By October 1647 there were no enemies of the Covenant in arms in Scotland. Decimated by the plague, ravaged by the destruction of crops, livestock and buildings, bankrupted by its military interventions in the other two kingdoms, and weakened by incessant bloodletting, Scotland was in a state of utter physical and moral exhaustion [34 (p. 311)].

In short, this period of turmoil arguably created the perfect ecological setting for the effective spread of an infectious disease such as the plague.

### 4. Public and local administrative responses to the pest

Given the extensive coverage of this issue by previous scholars we only provide a brief overview here. However, it is important to visit this issue, particularly as it speaks to the manner in which victims of the plague were dealt with (see following section). One overriding approach was to significantly restrict the movement of people within and between villages, towns, and cities. Oram [53 (p. 22)] notes that measures employed were more about preventing and containing outbreaks than anything else.

At a personal scale the following extract from the Peebles burgh records describes enforced isolation, which was a common approach to those suspected of carrying the Pest.

Thomas Tueddell wricht in Edinburgh being this day come furth of Edinburgh suspect of the plague of pestilence to his barne at the style callit Tueddellis style, being be the saidis proveist and bailyeis chargeit to remane within the said barne till sik tyme that the apperance of infectione be past, that the said Thomas Tueddell his wyfe nor nane of his servantes nor familie sail cum furth of the said barne for the space of fourtie dayes nixt heirefter, but sail remane within the said barne and hald the eister and southe doores thairof cloise steikit and lockit, and nawayes come furth thairof, nor haunt nor have companie or resort with any persones quhatsumeuir, nor suffer any resort to thame during the said space, and that vnder the pane of fyve hundreth pundis Scottes money and burning of the said berne incaice of failye, and the said James Halden to have the keiping the key of the southe doore of the said barne and be ansuerable thairfoir during that ilk tyme vnder the said pane, and that no meit nor drink sail be gevin in to thame outwith the presens of ane or tua of the counsell sessione or honest men of the burghe [54 (p. 379)].

[To summarise: Thomas Tuedell, his family, and servants, were ordered to quarantine in their home for 40 days and were not permitted to interact with anyone during this time. Failure to comply would mean a £500 (Scots) fine in addition to their home being burnt down. The key to their door was to be kept by a local official and they were not to receive any food or water without an official being present]

Such an approach also occurred on a much larger scale. Jillings [39 (p. 182)] details the extensive council efforts in 1647 of constructing and maintaining a quarantine facility on the links in Aberdeen. This amounted to a series of temporary wooden huts guarded by soldiers with the erection of gibbets on site apparently as a deterrent to escape, although Jillings notes the gibbets were never used for this purpose. This site was also employed for the mass burial of plague victims during this outbreak (see following section).

DesBrisay [55] in describing the immediate response to the pest outbreak in Aberdeen in 1647 relates how, apart from the usual cleaning activities (i.e., removal of rubbish middens) a watch of 120 armed men was deployed to limit ingress and egress from the town with a fine of £100 to any individual failing to take their turn at this task. Attempts by the populace to contact outsiders also incurred a £100 fine, while failure to report illness in one’s household attracted a £12 fine. Beggars were evicted from the city while a program of exterminating the non-human inhabitants (cats, dogs, rats, and mice) was instigated. Oram [53 (p. 16)] suggests the eradication of rats and mice was in response to an understanding of them as a vector of disease, although the eradication of all animals (including those that preyed on rodents) is more consistent with the vague and general belief in cleanliness being a deterrent to the disease, rather than a belief in the ability of animals to harbour and transmit the pest.

Sometimes, attempts at stopping the spread of plague led to unintended and catastrophic consequences. Hope [56 (p. 215)] notes that Kelso, on the borders, “hail housses, cornes, barnis, banyards, brunt be fyre, causit be a clinging [cleaning] off ane of the houses thairoff quhilk wes infecit with the plaig”. Apparently, Kelso was burnt down several times during the border wars [57 (p.584)] somewhat repeating an unfortunate pattern seen in the preceding 14^th^ and 15^th^ centuries, while this time the 700 left homeless were forced at gunpoint to stay put such was the fear of spreading the Pest [27 (p.140)].

### 5. Disposal of the dead

The Pest fundamentally altered both normative funerary rituals and the way victims of the plague were dealt with. These changes in funerary behaviour were arguably accompanied by a pervasive fear of the dead which was in part informed by their understanding of the cause(s) of infection. This section explores several ways in which the victims of plague were dealt with and the different motivations and, indeed, circumstances both victims and the survivors found themselves in. Was there actually a pervasive fear of the dead? Under what circumstances were mass burial pits employed? And, could victims be buried in church yards, and if yes, under what circumstances?

Kennedy [40 (Vol. 1, p. 271)] notes that plague victims in Aberdeen were buried “in the grounds near the windmill, and at the east end of the Castlehill, as well as in the Grayfriar’s croft. Each of these locations fall on the then east and northeastern periphery of New Aberdeen, with the windmill likely referring to a prominent named windmill on top of the highest of the three hills in the city (currently the north end of Gallowgate). East of Castlehill was the start of the links, while the Grayfriar’s croft likely refers to holdings the Franciscans owned behind the current Marischal College/Museum on Broad Street. However, the bulk of the dead were buried in trenches east of the rope works (a landmark present at the time Kennedy was writing) where the “[e]xpense of burying the dead, and for 37,000 turfs to cover over their graves, and carriage [amounted to] £153 6s 8 pence” [40 (Vol. 1, p. 271–272)]. Jillings [39 (p. 183)] provides a further break down of these costs, including the purchase of lime, and notes Robert Walker cut the turfs, Alexander Cruikschank and George Blaik transported them to site, while Alexander Deanes laid them. There was a belief that lime could, among other things, assist in the rapid decomposition of a body and suppress the smell, and associated disease carrying miasmas, associated with plague victims [58]. As far as we can determine, the site in question is the same as that excavated in both 1891 as part of a sewer extension, and a century later in 1987, providing three of the four individuals found positive for *Y*. *pestis*.

The use of plague pits was not uncommon in Scotland during this outbreak, or previous outbreaks for that matter. As noted previously, in 1647 some 600 individuals, or half of the population of Brechin, are reported to have died within a few months. “The most of them were buried in the little church-yard opposite to the porch door of the church…a part of them were buried in the large churchyard…” [59 (p. 127)]. A memorial was erected in the small kirk-yard with the following Latin inscription *Luna quarter crescens; Sexcentos peste peremptos; (Disce mori*!*) vidit; Pulvis et umbra fumus* (translated here): “In the span of four waxing moons / six hundred were struck down by plague / (Learn to die!) he saw / we are but dust and shadow” [59 (p. 127)]. This memorial was apparently replaced in 1869 with the same inscription [39 (p. 89)].

In Leith, where mortality was as high as 56%, there is archaeological evidence for the burial of 81 individuals that were either coffined, uncoffined or shrouded, on the Leith Links believed to be associated with temporary facilities to house the sick during the 1645 outbreak [40]. The observation of hastily constructed coffins, as well as some individuals remaining clothed and in possession of everyday items has been interpreted as indicating the bodies were not stripped of valuables thus indicating a fear of being infected by the dead [40 (p. 50)].

In Falkirk, Stirlingshire, those dying of the plague were interred on Graham’s Muir [Moor], with graves apparently covered with a flat stone with a stone wall erected around the cemetery [60 (p. 6–7)]. This suggests some degree of fear of the plague-dead and a need to keep the living distant from the dead, or perhaps to keep the dead from revisiting the living?

As a counter point to disposal of the dead in mass burials or plague pits is evidence for the continuity of more normative burial practices during plague outbreaks in general. On the 24^th^ of March 1647, less than two months prior to the cessation of council meetings in late March due to the Pest, the Aberdeen town council banned burial inside the structure of the Kirk of St Nicholas, although burial within kirks was not completely abolished until late 1649 [38]. While the plague has been reported as the primary catalyst for such changes in long-standing burial traditions, the conflicting Catholic and Presbyterian viewpoints about the importance of burial location cannot be ignored and indeed, the language used in this particular extract from the Council Register suggests more of an admonition for backsliding than anything else. Though burial inside churches was implicitly forbidden following the Reformation (c. 1559/60 in Aberdeen) this practice is not specifically referenced in either the First or Second Book of Discipline. Regardless of whether the 1647 ban was unquestioningly heeded, the council registers indicate that during the plague outbreak, members of the parish were buried on the south side and strangers (non-parish members) and the poor were buried on the north side of the kirkyard, with no mention made for any burial within the body of the kirk itself [38]. Though the burial records for the plague years have been lost (last seen in the 19^th^ century), Jillings [39 (p. 180)] notes that 71 plague victims were in fact buried in St Nicholas Kirkyard in the second half of 1647 while the burial registers for the parish of Old Machar (encompassing Old Aberdeen) records two burials of infected individuals within the churchyard.

Indeed, Spyrou and colleagues [61] analysed skeletal samples from numerous burial grounds across Europe where they identified *Y*. *pestis* from multiple types of graves including multiple burials in churchyards and single burials located in a chapter house in an Augustinian Friary. *Y*. *Pestis* has been identified in an individual buried in the churches of St Nicolay and St Clement in Oslo [62]. Similarly, *Y*. *pestis* has been found in normative burials in urban and rural cemeteries in Cambridge [63]. Clearly, some of these individuals must have been known to be victims of the Pest by their mourners whom, it would appear, may have been less afraid of contracting the plague from their dead as we might assume.

At an even smaller magnitude of scale, we have the intriguing normative treatment of two individuals buried on the banks of the Birns Water, a tributary of the River Tyne, on Gilchriston farm in East Lothian [44 (p. 310–11)]. A CANMORE record indicates the pair were put off a stagecoach travelling between London and Edinburgh as they showed signs of the plague [64]. Given the first fortnightly stagecoach journeys between these two cities was not established until 1658 [65 (p. 271)], it is unclear how likely this interpretation is. Martine [66] provides another interpretation, regarding William at least, whereby he was one of the last to perish from the plague in the area and was buried by one of his servants, a ploughman. Whatever the case, the biblical reference on the following grave inscription [66 (p. 36)] for William, suggests they were early victims of the mid-seventeenth century outbreak and were treated with some care in death.

*2 Sam., 24, David’s CHCIIE [Grief], Heir Lyes William Skirvin, Who Desicit the 24 of Ivinne [June?], 1645.

Heir Lyes Katrin Wilson Who Desicit, In Anno.

Flinn and colleagues [27 (p. 140–141)] refer to a very large gravestone in Kirkliston, the size and remote location they see as testament to the generally held superstitious fear of a resurgence of plague if such burials were disturbed. There are a number of entries regarding this grave in the Ordinance Survey Name Books for Mid-Lothian and while the details vary by entry the grave inscription is reported as “Here lies the dust of William Lin who died in the year of our Lord 1645” [67], with other entries suggesting he contracted the plague from his dog [68], was the last man who died of plague in Scotland [67], and his servant had to bury him as his neighbours had refused the task [67].

Fear seems to have been so great in some areas that those dying of the plague might not be buried at all. An anecdote from Tillicoultry concerning a man that had died suddenly, the assumption being from the plague, recounts that “the people were afraid to touch the corpse or even enter the house. It was pulled down, and the small eminence, which this occasioned, was called Botchy Cairn” [31 (p. 201)]. Similarly, McKerral [29 (p.75)] relays a story concerning the outbreak in Kintyre where green knolls on Kilkivan farm attest to the presence of houses that were left to decay and collapse on top of their occupants: plague victims. While fear of burying plague victims may have led to their abandonment, there also apparently a fear of improper burial to the extent that a further apocryphal anecdote for Highlanders to “order their coffins while still alive” [29 (p. 75)] to ensure proper burial. Finally, another story has it that a plague victim managed to convince friends to dig his grave, within which he lay until he died [29 (p. 75)].

Not all, and indeed only a fraction, of those that died from the Pest were afforded a church or bespoke burial. The question arises as to what conditions needed to be met to ensure a mass burial or individual interment? Criteria for mass burial likely includes the mortality rate for any given region and period, with death rates so high as to cause logistical issues in terms of normative burial likely resulting in industrial level interment. The presentation of symptoms, and potentially socio-economic status, likely also informed decisions for normal cemetery burial of plague victims. The bubonic form of plague leads to the obvious and visually striking development of buboes, or purulent abscesses, with mortality ranging between 40–70% [69 (p. 9)]. The pneumonic form, contracted through inhalation of the virus, presents as pneumonia which is fatal, although it can develop into the bubonic or septicaemic forms, the latter which is characterised by haemorrhagic and necrotic skin lesions while being lethal whatever the cause [69 (p. 9)]. It might be argued that the visually more confronting bubonic and septicaemic forms would have generated more fear in the community. Moreover, there was presumably some degree of variation in the severity of physical symptoms regardless of the form plague took, which may also speak to variability in attitudes to plague victims, some of whom may not necessarily have been recognised as having died of the plague.

Are we really seeing a fear of the dead, or fear of contracting plague from the dead? Normative burial treatment seems to belie either view. Further, plague pits or mass burial can simply be seen as an expedient manner with which to dispose of many corpses within a short period of time. The use of lime (e.g., Aberdeen plague pits on the Links), which both suppresses odours and from a late Medieval point of view suppresses disease carrying miasmas, may also be simply expedient when bodies are placed in a large open pit over an extended period. The possession of personal items by plague pit victims, as was demonstrated in Leith, may perhaps more parsimoniously be explained by logistical aspects, not least being the need for speedy burial of corpses, rather than a fear of the corpse or potentially contaminated possessions. In short, we are not seeing a homogenous approach to dealing with the dead during this outbreak, but rather one that was informed by a myriad of factors not necessarily uniformly driven by fear.

## Conclusions

This paper had several objectives with the first being to determine if *Yersinia pestis* was the pathogen involved in the last outbreak of plague in Aberdeen, and by implication Scotland as a whole, in the mid-17^th^ century. The aDNA analysis confirms the presence of *Y*. *pestis* in each of the three individuals tested, with evidence to suggest they were part of a mass burial on the Links in New Aberdeen that occurred in 1647/8. The geographic spread of the plague from 1644 through to 1649 is for the most part restricted to the central lowlands, a broad belt which is bounded by the Grampians to the north and the southern uplands to the south. Exceptions to this pattern include scattered occurrences up the east coast reaching as far as Peterhead in the northeast and an outbreak in the southwest on the Kintyre peninsula and Islay. The distribution of outbreaks most likely relates to a combination of population density (central lowland urban centres being hardest hit), transportation and trade networks (troop movements), in addition to the substantive disruption if not chaos caused by the civil war in general. It is unclear what role administrative responses, ostensibly employed to halt the advance and effects of the plague, had on a Scotland-wide scale. While cities such as Aberdeen had sophisticated plague prevention measures in place, its distance from the central plague belt in the central lowlands was perhaps a more important factor. Finally, despite the ravages of war, economic hardship, and the additional complications of a terrible epidemic, normative burial practices continued to be employed throughout this final Scottish epidemic. It is unclear if a general fear of the dead and contracting the Pest from plague victims can be used to characterise mid-17^th^ century Scottish public opinion. Arguably, plague pits and mass burials in general were more a practical response to the logistical difficulties associated with disposing of the dead on a large scale. Having the plague was not a one-way ticket to a plague pit and, indeed, there are many examples of plague victims receiving normative and caring burial treatment despite any potential risks to the bereaved.

## Supporting information

S1 File Supporting information. (DOCX)
