# Correction: The final plague outbreak in Scotland 1644–1649: Historical, archaeological, and genetic evidence

**DOI:** 10.1371/journal.pone.0317062

**Published:** 2025-01-02

**Authors:** Jenna Dittmar, Rebecca Crozier, Toni de-Dios, Christiana L. Scheib, Jackson W. Armstrong, Jenny Pape, Ross MacLennan, Ricky Craig, Marc Oxenham

The following information is missing from the Acknowledgments section: The authors wish to express their sincere gratitude to Stine M. Carlsson and all of the members of the 14Chrono Centre at Queen’s University Belfast for generating the radiocarbon dates provided in Table 1 (ABDMS 23509.1, Lab # UBA47632; 23509.3, UBA47634; 23509.4, UBA47633).
