# Supplementary material for: The final plague outbreak in Scotland 1644–1649: Historical, archaeological, and genetic evidence
Source: PLoS One. 2024 Nov 13;19(11):e0306432. doi: 10.1371/journal.pone.0306432 (PMC11559987; doi:10.1371/journal.pone.0306432)
Supplement: S1 File — (DOCX) [file pone.0306432.s001.docx]

**Supplementary Information**

| Museum No. ABDMS | Laboratory ID | No. of read pairs sequenced |
| --- | --- | --- |
| 023509.1 | YYY054 | 7,589,704 |
| 023509.2 | YYY055 | 7,418,530 |
| 023509.3 | YYY056 | 7,550,364 |

SI Additional Sample Information.


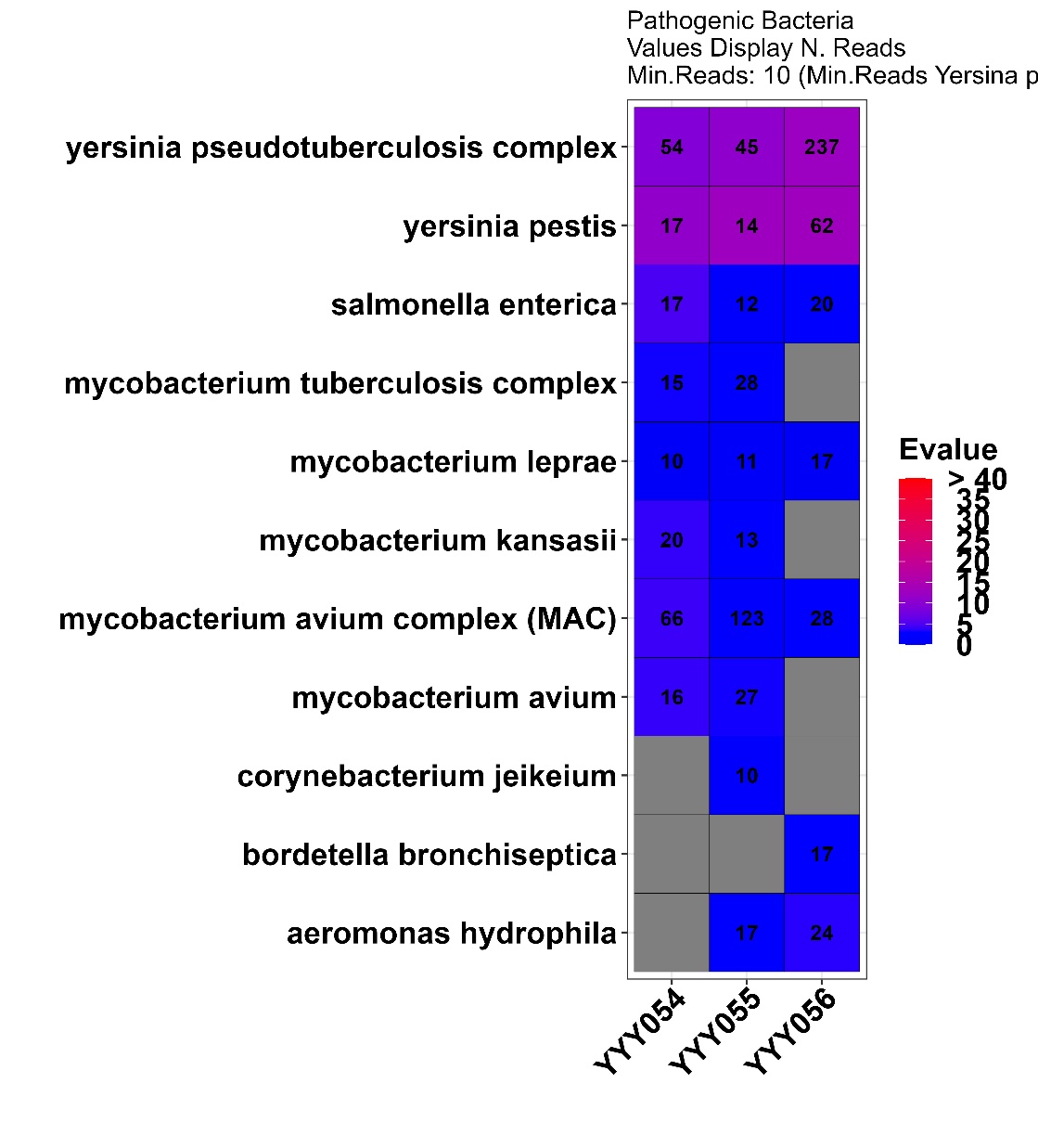


SI Metagenomic screening results. [Results of the metagenomic screening performed using KrakenUniq. The colour indicates the E-value, the higher the value is, the most probable the hit is reliable. The number of sequences matching a specific taxon is indicated inside the cell. E-value is not displayed if the number of sequences is below 10 (5 in the case of *Y. pestis*)].

| Species | Strain | Accession | Publication Reference |
| --- | --- | --- | --- |
| *Yersinia pestis* | *CO92* | ASM906v1 | [1] |
| *Yersinia pseudotuberculosis* | *IP32953* | ASM83429v1 | [2] |

SI Reference Genomes Used. [Accession codes and strains of the reference genomes used to map against *Yersinia pestis* and *Yersinia pseudotuberculosis*].


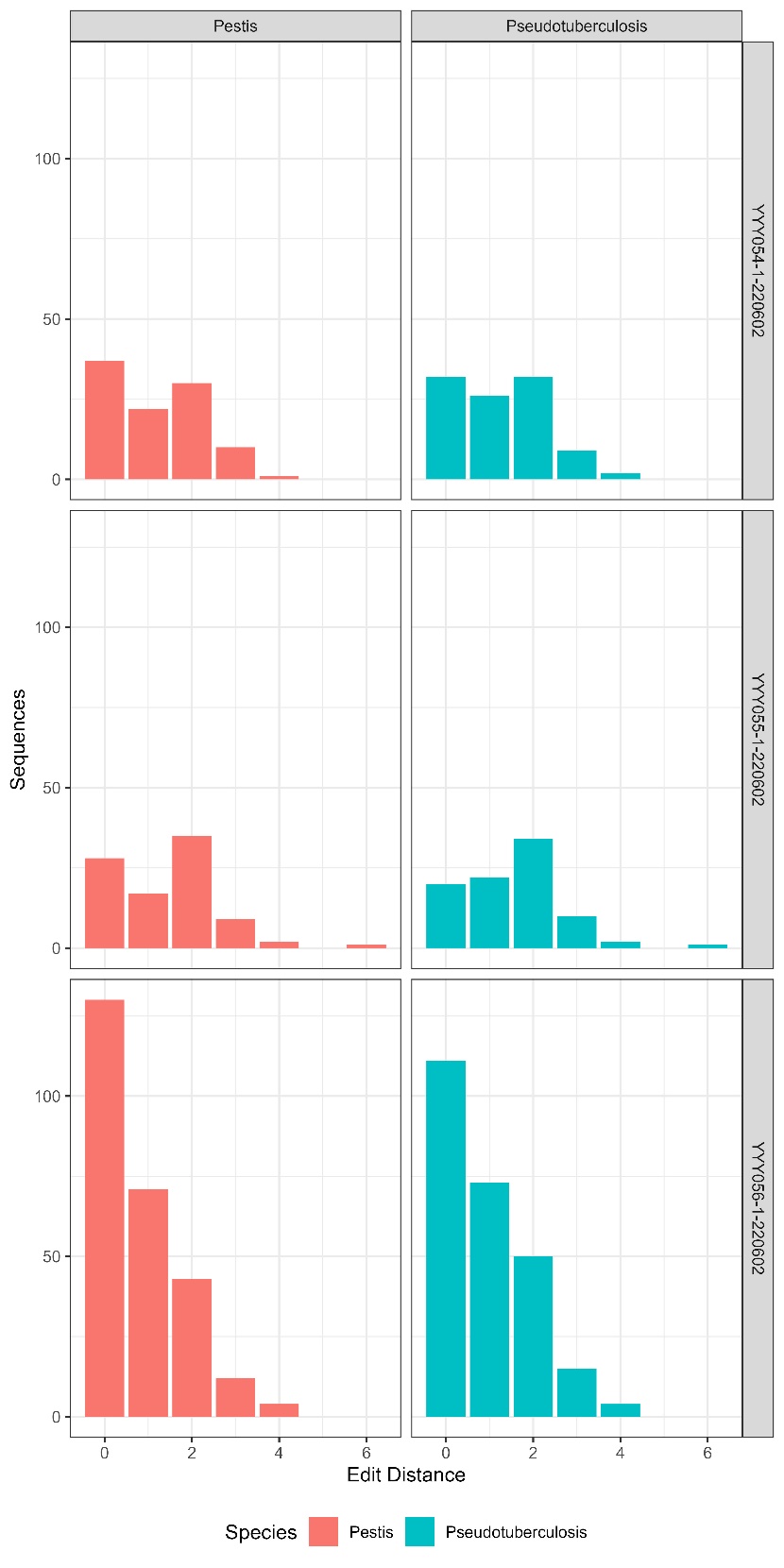


SI Edit Distance comparison between *Yersinia pestis* and *Yersinia tuberculosis*. [Edit distance distribution of quality 30 reads mapping against *Y. pestis* and *Y. tuberculosis* reference genome per sample. Higher values denote more genetic distance, while 0 is indicative of identical matching].


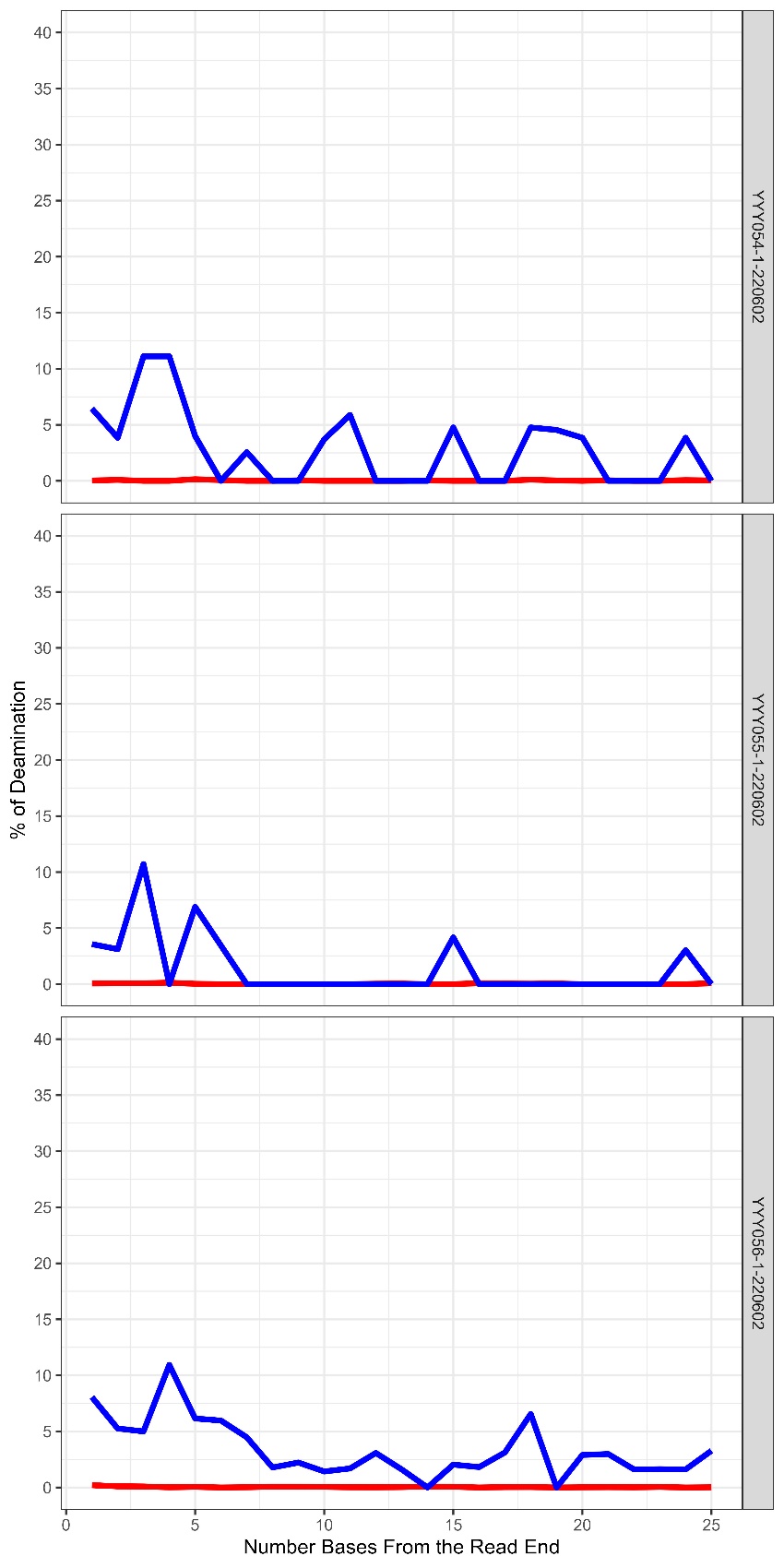


SI aDNA damage patterns. [Percentage of deamination at sequences’ end associated to post-mortem damage].

**Supplementary References**

1.Parkhill J, Wren BW, Thomson NR, Titball RW, Holden MTG, Prentice MB, Sebaihia M, James KD, Churcher C, Mungall KL, Baker S, Basham D, Bentley SD, Brooks K, Cerdẽo-Tárraga AM, Chillingworth T, Cronin A, Davies RM, Davis P, Dougan G, Feltwell T, Hamlin N, Holroyd S, Jagels K, Karlyshev AV, Leather S, Moule S, Oyston PCF, Quail M, Rutherford K, Simmonds M, Skelton J, Stevens K, Whitehead S, Barrell BG. Genome sequence of Yersinia pestis, the causative agent of plague. 2001; Nature 413, 523–527. https://doi.org/10.1038/35097083.

2.Johnson SL, Daligault HE, Davenport KW, Jaissle J, Frey KG, Ladner JT, Broomall SM, Bishop-Lilly KA, Bruce DC, Coyne SR, Gibbons HS, Lo CC, Munk AC, Rosenzweig CN, Koroleva GI, Palacios GF, Redden CL, Xu Y, Minogue TD, Chain PS. Thirty-two complete genome assemblies of nine Yersinia species, including Y. pestis, Y. pseudotuberculosis, and Y. enterocolitica. 2016; Genome Announc. 3. https://doi.org/10.1128/genomeA.00148-15.
